# Intrinsic Curvature-Induced Regulation of Interfacial Thermal Transport in Janus TMD Heterostructures

**DOI:** 10.3390/molecules31162876

**Published:** 2026-08-18

**Authors:** Lixia Shi, Lei Huang, Liqi Xiong, Jianping Li

**Affiliations:** 1 School of Intelligent Manufacturing, Changzhou Vocational Institute of Textile and Garment, Changzhou 213164, China; 2 School of Mechanical Engineering, Southeast University, Nanjing 211189, China; 3 School of Mechanical and Electronic Engineering, Nanjing Forestry University, Nanjing 210037, China; 4 School of Automotive & Transportation Engineering, Shenzhen Polytechnic University, Shenzhen 518055, China

**Keywords:** Janus TMDs, intrinsic curved interface, interfacial thermal conductance, phonon transport, nonequilibrium molecular dynamics

## Abstract

Efficient interfacial thermal transport in two-dimensional heterostructures is essential for improving heat dissipation and operational stability in next-generation energy conversion and energy electronic devices. Here, nonequilibrium molecular dynamics simulations are employed to investigate the thermal transport behaviors of WSSe/MoS_2_ and WSSe/MoSe_2_ lateral heterostructures. Unlike the commonly assumed flat interface, both heterostructures spontaneously form an intrinsically curved interface after relaxation, introducing a unique structural feature for phonon transport. The armchair interface exhibits higher interfacial thermal conductance than the zigzag counterpart due to stronger phonon coupling. Furthermore, external strain can effectively regulate thermal transport through the competition between interface flattening and phonon scattering, while increasing temperature enhances thermal conductance by activating low-frequency phonons. In contrast, vacancy defects also significantly suppress heat transfer by disrupting interfacial bonding. This work reveals the critical role of intrinsic interface curvature in phonon-mediated thermal transport and provides a new strategy for designing high-performance thermal management materials based on Janus heterostructures for advanced energy conversion and storage technologies.

## 1. Introduction

Two-dimensional (2D) materials have attracted extensive attention as promising candidates for next-generation nanoelectronic [1], optoelectronic [2] and energy-conversion devices [3] owing to their atomically thin structures [4], tunable electronic properties [5,6] and exceptional thermal characteristics [7]. As device dimensions continue to shrink, heat dissipation has emerged as one of the primary factors limiting device reliability and performance [8]. In low-dimensional systems, thermal energy is predominantly transported by phonons rather than free electrons, making phonon dynamics the governing mechanism of heat conduction [9]. Unlike bulk materials, the thermal transport behavior of 2D materials is strongly influenced by lattice symmetry [10], anharmonic phonon scattering [11] and dimensional confinement effects [12]. Consequently, understanding phonon-mediated heat transport has become a central topic in thermal management research [13]. Considerable efforts have been devoted to exploring thermal conductivity in graphene [14], hexagonal boron nitride (h-BN) [15], phosphorene [16] and transition metal dichalcogenides (TMDs) [17]. Graphene exhibits an ultrahigh thermal conductivity exceeding 3000 W m^−1^ K^−1^ due to its long phonon mean free path and strong *sp*^2^ covalent bonding [18]. However, the absence of an intrinsic bandgap limits its practical application in semiconductor devices [19]. In contrast, semiconducting TMDs provide an attractive balance between electronic functionality and thermal transport performance, rendering them highly promising for integrated thermal-management applications [20]. Therefore, elucidating the phonon transport mechanisms in emerging 2D semiconductors is of both fundamental and technological significance [21].

Recently, 2D heterostructures have been recognized as an effective strategy for tailoring thermal transport properties through interface engineering [22]. In these systems, interfacial thermal conductance (ITC) is primarily governed by phonon transmission, phonon spectral matching, interfacial bonding strength, and lattice mismatch [23]. Previous studies have demonstrated that interfacial configuration, strain, defect distribution, and temperature can significantly influence phonon coupling and heat transfer efficiency across heterogeneous interfaces [24,25]. Among various 2D heterostructures, TMDs have attracted particular interest because of their structural compatibility, chemical stability, and tunable electronic properties [26,27]. More recently, Janus TMDs, represented by MoSSe and WSSe, have emerged as a new class of asymmetric 2D materials in which one chalcogen layer is replaced by another, resulting in intrinsic out-of-plane dipole moments, broken mirror symmetry, enhanced piezoelectricity, and unique phonon transport characteristics [28,29,30]. These distinctive features provide additional degrees of freedom for manipulating interfacial heat transport compared with conventional TMDs. Furthermore, the successful experimental synthesis of Janus MoSSe/WSSe lateral heterostructures has opened new opportunities for investigating thermal transport across atomically sharp interfaces [31]. Nevertheless, the effects of interface orientation, atomic termination, strain engineering, and temperature on phonon transmission and interfacial thermal conductance in Janus-TMD lateral heterostructures remain insufficiently understood. Additionally, 2D heterostructures are not perfectly planar under realistic conditions, but often exhibit corrugations and wrinkles, which induce local strain fields and significantly in-fluence their mechanical, thermal and catalytic properties [32,33]. Experimentally, wrinkled 2D TMDs can be artificially fabricated using nanocone substrates, offering new possibilities for the design of thermal management materials [34]. However, most previous studies have focused on the effects of externally induced wrinkles or buckling structures on electronic and mechanical properties [35], while their influence on interfacial thermal transport remains largely unexplored. Unlike these extrinsic deformations generated by substrate mismatch, thermal expansion or mechanical loading, the intrinsic curvature originates from the inherent structural asymmetry of the heterostructure and does not require external strain. This intrinsic geometric feature provides a unique pathway to regulate phonon coupling and interfacial thermal transport, offering a fundamentally different strategy for thermal management.

In this work, we investigate the effect of intrinsic curved interfaces on interfacial thermal transport in Janus TMD heterostructures through atomic-scale simulations and phonon analysis. Unlike previous studies mainly focusing on planar interfaces, we reveal the relationship between intrinsic interface curvature, structural characteristics and phonon transport behavior, demonstrating that intrinsic curved interfaces can serve as an effective strategy for regulating interfacial thermal transport. This study provides new insights into phonon engineering and the design of advanced thermal management materials.

## 2. Computational Methods

**Figure 1:**
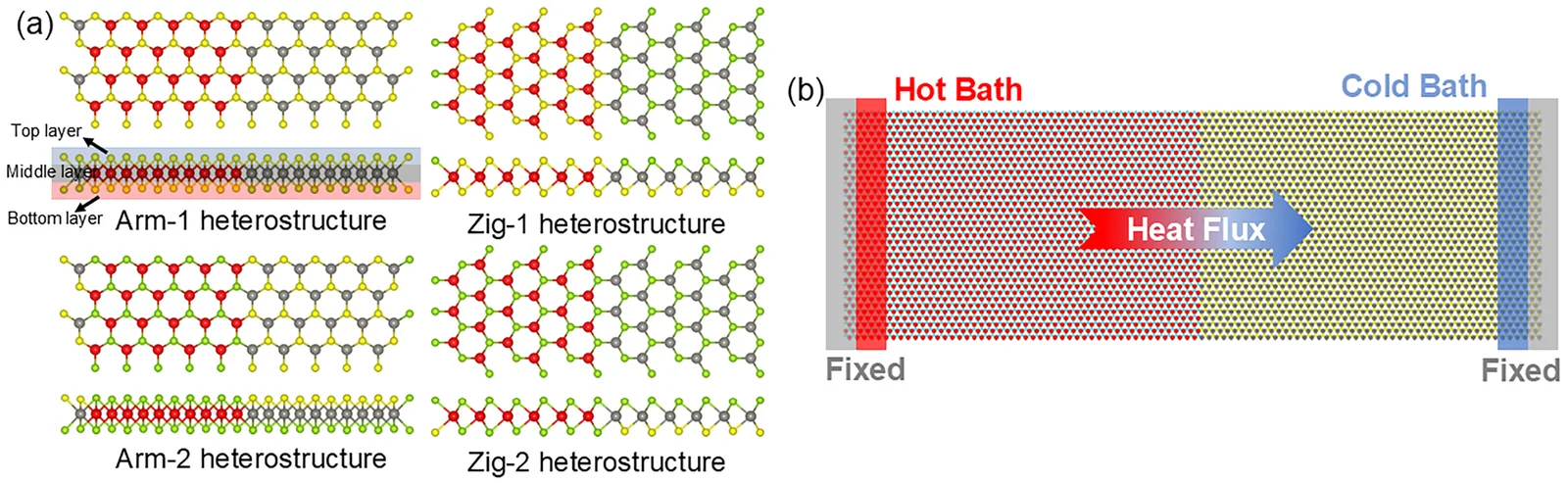
(**a**) Top and side views of the four constructed lateral WSSe/MoX_2_ heterostructures, including Arm-1, Arm-2, Zig-1, and Zig-2 configurations. Red, gray, yellow, and cyan atoms denote Mo, W, S and Se, respectively. (**b**) Schematic representation of the NEMD simulation model. The left and right ends of the heterostructure are coupled to hot and cold thermal reservoirs, respectively, to induce a steady heat flux across the interface. Fixed boundary conditions are imposed at both ends of the simulation cell.

The atomic models of the lateral WSSe/MoX_2_ (X = S, Se) heterostructures are shown in Figure 1a. To investigate the influence of interface orientation and atomic termination on thermal transport, four representative heterostructures, namely Arm-1, Arm-2, Zig-1 and Zig-2, were constructed by laterally stitching monolayer MoX_2_ along the armchair and zigzag directions. Specifically, Arm-1 (or Arm-2) and Zig-1 (or Zig-2) correspond to WSSe/MoS_2_ (or WSSe/MoSe_2_) heterostructure with armchair (or zigzag) configuration along the interfaces. In the structural models, red, gray, yellow, and cyan atoms denote Mo, W, S, and Se atoms, respectively. Notably, a recent experimental study has successfully synthesized a structurally analogous lateral MoSSe/WSSe heterostructure with a zigzag interface via chemical vapor deposition (CVD), demonstrating the experimental feasibility of such Janus-based lateral heterostructures [31].

In our molecular dynamics (MD) simulations, the dimensions of the WSSe/MoX_2_ heterostructure with armchair and zigzag interface, named as Armchair and Zigzag heterostructure, were 303.08 Å × 99.464 Å and 303.92 Å × 98.90 Å, respectively. To evaluate the interfacial thermal transport properties, non-equilibrium molecular dynamics (NEMD) simulations [36] were performed using the model illustrated in Figure 1b. The heterostructure was divided into three regions along the heat transport direction, including a hot bath, a transport region, and a cold bath. To suppress atomic evaporation and maintain structural stability, atoms located at both ends of the simulation cell along the *x*-direction were fixed. Adjacent atomic layers were coupled to Nosé–Hoover thermostats [37], serving as thermal reservoirs. Heat was continuously injected into the hot reservoir and extracted from the cold reservoir to establish a steady-state temperature gradient across the system, resulting in a net heat flux from the hot side to the cold side. Fixed boundary conditions were applied at both ends of the simulation cell to eliminate rigid-body motion and ensure simulation stability. To investigate the heat transfer characteristics of the heterostructure at 300 K, the temperature of the hot and cold bath is set as 320 K and 280 K, respectively. Then, under steady-state conditions, the temperature profile and heat flux were employed to calculate the interfacial thermal conductance of the WSSe/MoX_2_ heterostructures, thereby revealing the effects of interface configuration on phonon-mediated heat transport.

All our MD simulations were carried out using the Large-scale Atomic/Molecular Massively Parallel Simulator (LAMMPS) [38]. Structural configurations and atomic trajectories were visualized with the Open Visualization Tool (OVITO) [39]. The interatomic interactions within the WSSe/MoX_2_ lateral heterostructure were described by the Stillinger–Weber (SW) potential, whose parameters were derived from the valence force field (VFF) model [40]. The reliability of this potential has been validated by comparing the calculated phonon dispersion of monolayer MoS_2_ with experimental measurements, where both the VFF model and the corresponding SW potential successfully reproduce the experimentally observed phonon branches [41]. Moreover, this SW potential framework has been successfully applied to describe cross-species interactions in multi-component TMDs heterostructures, including W–Mo and Se–S interactions, and has been validated in studies of their mechanical and thermal transport properties [9,26,42]. Therefore, the SW potential provides a reliable description of lattice dynamics and phonon transport in Janus TMD systems. Consequently, the SW potential adopted in the present work provides a reliable framework for describing the atomic dynamics of Janus TMDs. Moreover, this potential has been extensively employed in simulations of mechanical deformation, including uniaxial tension and strain engineering, as well as thermal transport studies [43,44]. To ensure computational accuracy and energy conservation throughout the simulations, the integration time step is fixed at 1.0 fs. Thermal transport properties were evaluated using the NEMD approach, with heat transfer imposed normal to the interface of the lateral heterostructure [45]. Regarding the boundary treatment, non-periodic boundary conditions were imposed along the *x*-direction to facilitate the formation of a stable temperature gradient. Periodic boundary conditions were applied along the in-plane *y*-direction, which is perpendicular to the heat-flow direction, thereby minimizing finite-size and edge effects while representing an infinitely extended sheet. Along the out-of-plane *z*-direction, a sufficiently large vacuum region combined with fixed boundary conditions was introduced to eliminate spurious interactions between adjacent periodic images. Then, all systems were initially relaxed within the *NPT* (isothermal–isobaric) ensemble for 100 ps to achieve appropriate density and pressure equilibration. This was followed by further relaxation under the *NVT* (canonical) ensemble using a Nosé–Hoover thermostat for 2000 ps to stabilize the temperature. Subsequently, the systems were switched to the *NVE* (microcanonical) ensemble for an additional 2000 ps to eliminate residual thermostat effects and ensure energy conservation. Throughout the equilibration process, both total energy and temperature exhibited small fluctuations around steady mean values, confirming that thermodynamic equilibrium had been reached. To establish a nonequilibrium steady state, NEMD simulations were then performed for 24 ns until both the temperature gradient and heat flux became time-independent. The heat flux (*J*) was calculated as follows [46]:

(1) $$J=\frac{1}{V}\left[\sum_{i}^{N}\varepsilon_{i}v_{i}+\frac{1}{2}\sum_{ij;i\neq j}^{N}\left(F_{ij}\cdot v_{i}\right)r_{ij}\right.\left.+\frac{1}{6}\sum_{ijk,i\neq j\neq k}^{N}\left(F_{ijk}\cdot v_{i}\right)\left(r_{ij}+r_{ik}\right)\right],$$

where *E* represents the system energy, while *v*_i_ denotes the velocity of atom *i*. *ε*_i_ and *v*_i_ represent the energy and velocity of atom *i*. The term *r_ij_* corresponds to the interatomic distance between atoms *i* and *j*. The two-body and three-body interaction forces are described by *F_ij_* and *F_ijk_*, respectively. *V* refers to the total volume of the investigated system. The interfacial thermal conductance (*G*) of the studied heterostructure is calculated as follows [47]:

(2) $$G=\frac{J}{\Delta T},$$

where *J* and the Δ*T* are used to represent the heat flux and the temperature jump across the interface of the WSSe/MoX_2_ heterostructures. The vibrational density of states (VDOS) is obtained by the following [48]:

(3) $$VDOS(\omega )=\frac{1}{\sqrt{2\pi }}\int_{0}^{\infty }e^{i\omega T}C\left(t\right)dt,$$

where *ω* and *C*(*t*) express the angular frequency and the velocity autocorrelation function, respectively, and where *C*(*t*) is defined as $C\left(t\right)=\left\langle \sum_{j=1}^{N}\bar{\nu_{j}}\left(0\right)\cdot {\bar{v}}_{j}\left(t\right)\right\rangle$. The ${\bar{v}}_{j}\left(t\right)$ demonstrates the velocity of atom *j* and the ⟨ ⟩ is the ensemble average.

## 3. Results and Discussion

**Figure 2:**
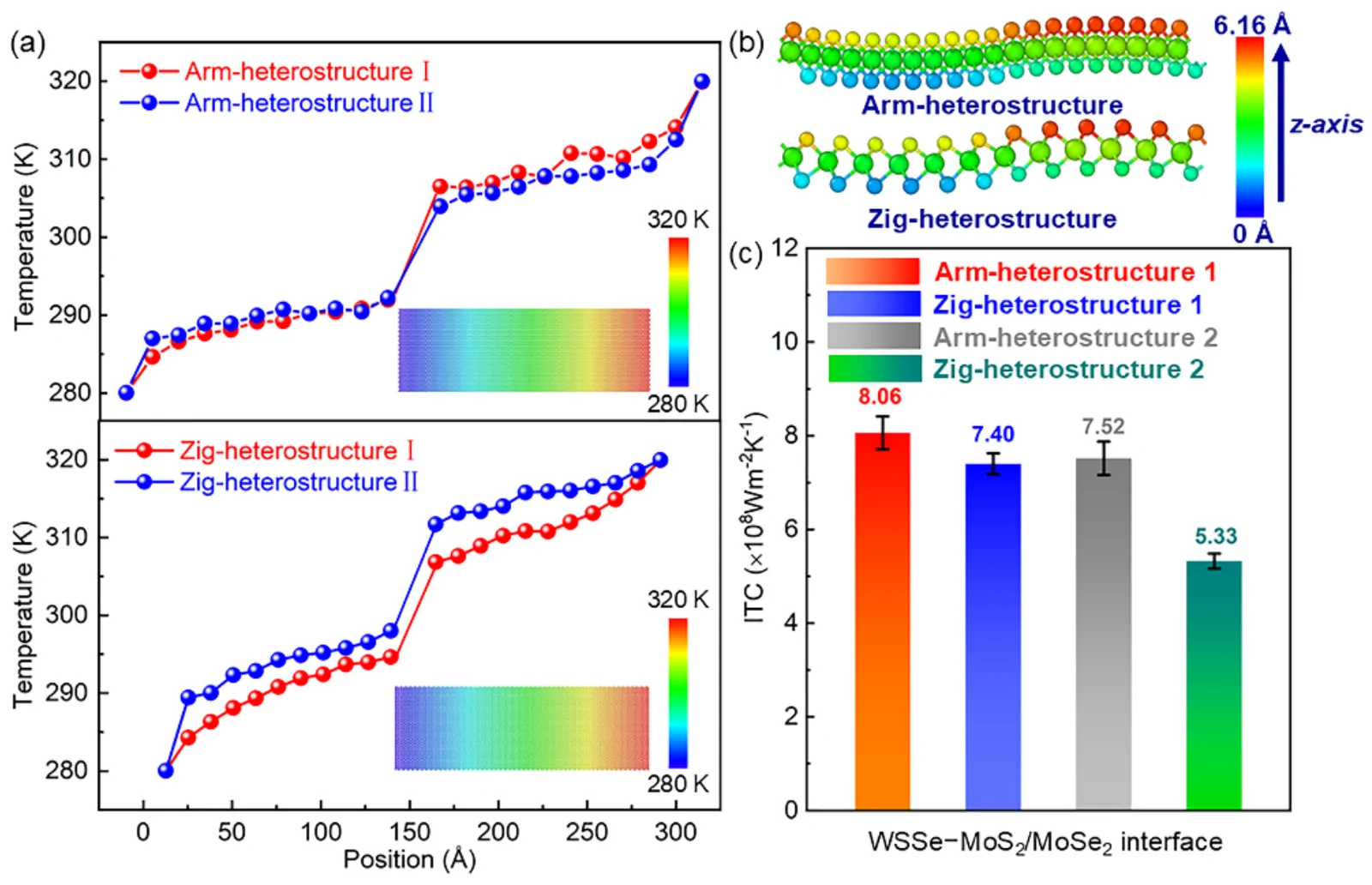
(**a**) The calculated temperature distribution of the WSSe/MoX_2_ heterostructures. (**b**) The relaxed structures of the Armchair and Zigzag WSSe/MoX_2_ heterostructures; the color contour indicates the atomic *z*-coordinate. (**c**) The NEMD obtained interfacial thermal conductance of the WSSe/MoX_2_ heterostructures along the heat flow direction.

After full equilibration, the WSSe/MoX_2_ heterostructures can maintain their structural configuration without noticeable distortion, indicating robust thermal stability at 300 K. And the steady-state temperature profiles along the heat flow direction exhibit clear interfacial temperature discontinuities for all configurations, as shown in Figure 2a, indicating the existence of finite interfacial thermal resistance at the WSSe/MoX_2_ interfaces [49]. A pronounced temperature jump is observed near the heterointerface region, while nearly linear temperature gradients are maintained within each constituent layer, confirming diffusive phonon transport in the WSSe and MoX_2_ monolayers. It is worth noting that Δ*T* was determined through linear fitting and extrapolation to reduce the uncertainty arising from direct measurements [50]. Notably, the magnitude of the interfacial temperature drop differs significantly between Armchair and Zigzag heterostructures, suggesting a strong dependence of thermal transport on interfacial atomic registry and edge chirality. Interestingly, both the Armchair and Zigzag WSSe/MoS_2_ and WSSe/MoSe_2_ heterostructures spontaneously evolve into an intrinsically curved interfacial configuration, rather than remaining atomically flat after full structural relaxation, as shown in Figure 2b. This intrinsic bending originates from the asymmetric interfacial torque induced by the structural asymmetry of the Janus WSSe layer and the lattice mismatch between the constituent monolayers, which drives out-of-plane deformation to minimize the total energy of the heterostructure. Since the interfacial morphology plays a crucial role in determining phonon transmission across the interface, the formation of such an intrinsic curved interface is expected to significantly influence the interfacial thermal transport, thereby motivating a systematic investigation of its thermal transport characteristics. Unlike the conventionally assumed atomically flat interfaces, the spontaneously formed intrinsic curved interface introduces an additional structural degree of freedom for phonon transport. Therefore, understanding how this unique interfacial morphology affects heat transfer is essential for revealing the thermal transport mechanism of Janus-based heterostructures.

Thus, as summarized in Figure 2c, the calculated interfacial thermal conductance of these heterostructures follows the trend: Arm-1 heterostructure (8.06 × 10^8^ W m^−2^ K^−1^) > Arm-2 heterostructure (7.52 × 10^8^ W m^−2^ K^−1^) and Zig-1 heterostructure (7.40 × 10^8^ W m^−2^ K^−1^) > Zig-2 heterostructure (5.33 × 10^8^ W m^−2^ K^−1^). One can see that the ITC of the Armchair heterostructure is higher than that of the Zigzag one. This pronounced anisotropy can be attributed to the distinct interfacial phonon transmission pathways governed by stacking geometry [26]. In the armchair configuration, better atomic matching and enhanced vibrational coupling facilitate more continuous phonon transport channels across the interface, reducing phonon scattering and suppressing localized vibrational states. In contrast, the zigzag interface introduces stronger lattice mismatch and more pronounced interfacial disorder, which enhances phonon reflection and mode conversion, thereby reducing the effective phonon transmission coefficient. Furthermore, variations between WSSe/MoS_2_ and WSSe/MoSe_2_ heterostructures suggest that subtle differences in stacking sequence further modulate interfacial bonding strength and phonon spectral overlap, ultimately governing the observed thermal transport efficiency.

**Figure 3:**
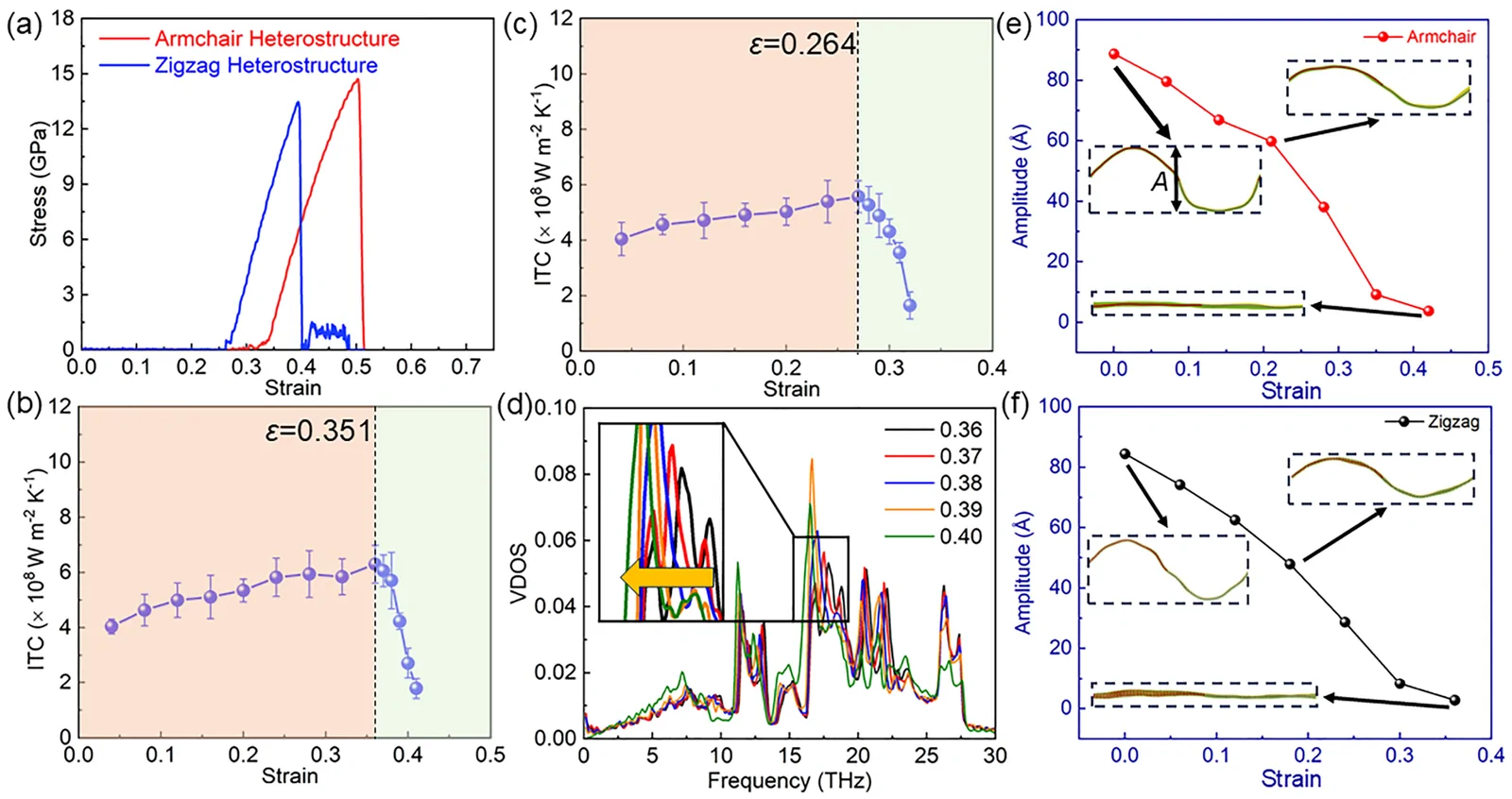
(**a**) The obtained stress−strain behaviors of the Armchair and Zigzag WSSe/MoS_2_ heterostructures. The calculated interfacial thermal conductance under different strain for (**b**) Armchair WSSe/MoS_2_ and (**c**) Zigzag WSSe/MoS_2_ heterostructures. (**d**) The obtained vibrational dynamic density of states of the Armchair WSSe/MoS_2_ heterostructure under different strains. The amplitude of bending (**e**) Armchair WSSe/MoS_2_ and (**f**) Zigzag WSSe/MoS_2_ heterostructures under different external strain.

Mechanical–thermal coupling at the interface has emerged as an effective strategy for regulating the interfacial thermal conductance of heterostructures [51]. Given the unique characteristics of such intrinsically curved interfaces, investigating the response of interfacial thermal conductance to externally applied mechanical strain is particularly meaningful and deserves further exploration. Therefore, the mechanical response and interfacial thermal transport behaviors of Armchair and Zigzag WSSe/MoS_2_ heterostructures under uniaxial tensile strain are addressed. In Figure 3a, one can see that both heterostructures exhibit an initial linear elastic regime followed by nonlinear deformation and eventual fracture. The Armchair heterostructure demonstrates a higher fracture strain (about 0.50) and tensile strength (about 14.5 GPa) compared with the Zigzag heterostructure, which fails at a lower strain (about 0.40) with a tensile strength of approximately 13 GPa. This anisotropic mechanical behavior originates from the distinct atomic arrangements along the armchair and zigzag directions, resulting in different bond-stretching mechanisms and load-transfer efficiencies under tensile loading. The higher ductility of the Armchair heterostructure indicates a greater ability to accommodate lattice deformation before structural failure. The variation in interfacial thermal conductance with strain for the Armchair heterostructure is illustrated in Figure 3b that the ITC initially increases from approximately 4.0 × 10^8^ to 6.2 × 10^8^ W m^−2^ K^−1^ as the strain increases to *ε* = 0.351. This enhancement can be attributed to strain-induced modulation of the interfacial phonon spectrum, which improves phonon coupling across the WSSe/MoS_2_ interface and facilitates more efficient energy transfer. However, when the applied strain exceeds the critical value of *ε* = 0.351, the ITC decreases sharply. At large strains, significant lattice distortion weakens interfacial atomic interactions and increases phonon scattering, thereby suppressing heat transport across the interface. This behavior suggests the existence of an optimal strain window for maximizing thermal transport performance. A similar trend is observed for the Zigzag heterostructure, as shown in Figure 3c. The ITC gradually increases with increasing strain and reaches a maximum value near *ε* = 0.264, after which a pronounced decline occurs. Notably, the critical strain corresponding to the maximum ITC is substantially lower than that of the Armchair heterostructure, indicating that the Zigzag configuration is more sensitive to tensile deformation. The earlier onset of ITC degradation is consistent with its lower mechanical stability and reduced ability to preserve effective interfacial phonon transmission under large lattice distortions [52]. It is worth noting that the strain is applied quasi-statically, with full structural relaxation performed at each strain increment. No bond breaking or structural instability is observed within the investigated strain range. Additionally, a previous study has demonstrated that the asymmetrical structure induced curved interface can fully recover its original bending morphology after mechanical straightening and subsequent stress release. This reversible mechanical response also provides opportunities for the development of adaptive catalysts [3]. Therefore, the strain-dependent variation in ITC is attributed to the reversible modulation of phonon transport rather than irreversible structural changes.

To further investigate the microscopic mechanism governing the strain-dependent thermal transport, Figure 3d presents the vibrational density of states of the Armchair heterostructure under different tensile strains. As the strain increases from 0.36 to 0.40, the low-frequency phonon peaks exhibit a noticeable shift toward lower frequencies, accompanied by peak broadening and intensity variation. Such phonon softening indicates a reduction in bond stiffness induced by tensile deformation. Near the critical strain region, the overlap between vibrational modes on both sides of the interface becomes enhanced, promoting phonon transmission and leading to the observed increase in ITC [53]. However, further strain application causes excessive lattice distortion, resulting in weakened phonon coherence and increased anharmonic scattering. Consequently, the spectral matching between the two constituent layers deteriorates, reducing interfacial phonon transport efficiency and causing the rapid decline in ITC. These results demonstrate that strain engineering can effectively regulate interfacial heat transfer by altering the phonon vibrational characteristics and coupling strength at the WSSe/MoS_2_ interface.

To quantitatively characterize the evolution of the intrinsic curved interface under external tensile strain, the bending amplitude (*A*) of the Armchair and Zigzag WSSe/MoS_2_ heterostructures is extracted and presented in Figure 3e and f, respectively. For both interface orientations, the bending amplitude decreases progressively with increasing tensile strain, indicating that external stretching effectively suppresses the intrinsic curvature and drives the interface toward a flatter configuration. The Armchair heterostructure exhibits a relatively gradual reduction in *A*, whereas the Zigzag configuration shows a more pronounced response to tensile deformation, consistent with its lower mechanical stability. At sufficiently large strain, the bending amplitude approaches a nearly zero value, corresponding to an almost flattened interface. This strain-induced flattening improves the geometric continuity across the interface and facilitates more direct phonon transmission, which is consistent with the initial increase in ITC observed in Figure 3b,c. However, further tensile deformation beyond the optimal strain range introduces substantial lattice distortion and enhanced phonon scattering, resulting in a decrease in ITC despite the continued reduction in bending amplitude. These results demonstrate that external strain provides an effective means of continuously regulating the intrinsic curvature of Janus heterostructures, with the resulting competition between interface flattening and strain-induced phonon scattering governing the thermal transport response.

**Figure 4:**
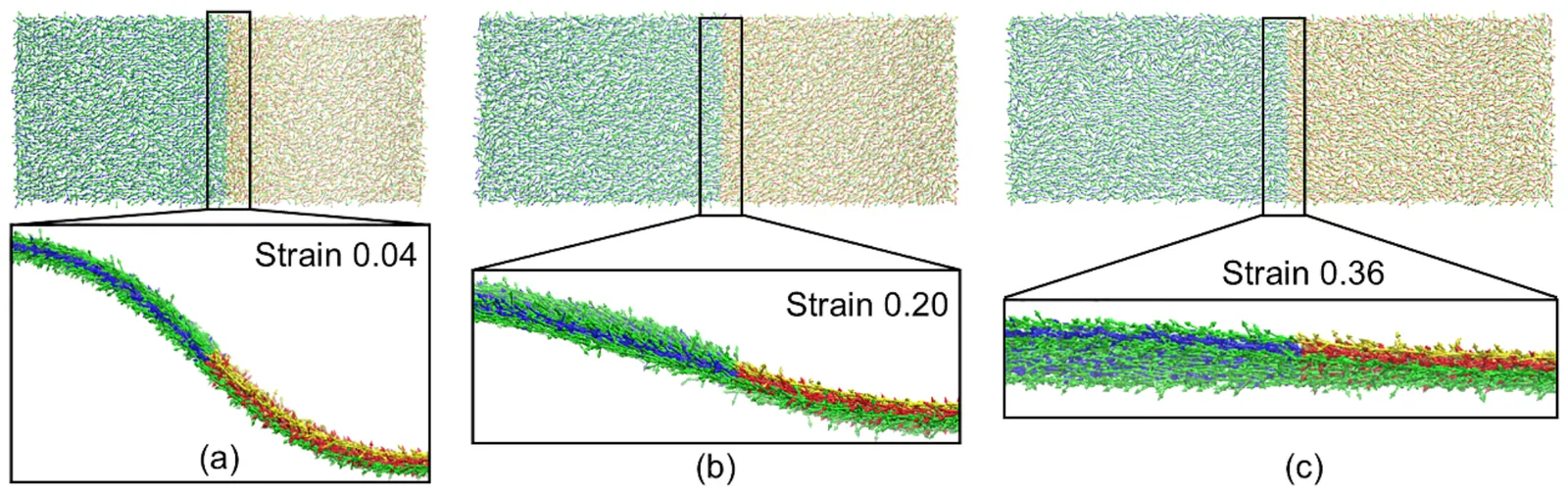
The calculated top view and locally magnified side view of the atomic heat flux distribution under strains of (**a**) 0.04, (**b**) 0.20 and (**c**) 0.36.

Then, the heat flux distributions of the WSSe/MoS_2_ heterostructure are obtained at representative tensile strains of 0.04, 0.20, and 0.36 to provide microscopic insight into the evolution of interfacial heat transport. At a low strain of 0.04 (Figure 4a), the intrinsic curved interface causes noticeable deflection of the heat flux vectors near the interface. The non-uniform heat flow indicates that local geometric distortion interrupts continuous phonon transport, resulting in additional phonon scattering and a relatively large interfacial thermal resistance. As the tensile strain increases to 0.20, shown as Figure 4b, the interfacial curvature is gradually reduced and the interface becomes smoother. Consequently, the heat flux vectors become more aligned across the interface, suggesting a more continuous heat-transfer pathway with reduced phonon scattering. This improved heat-flow continuity is consistent with the gradual increase in interfacial thermal conductance. When the strain further increases to 0.36, in Figure 4c, the interface has been almost completely flattened, and the heat flux exhibits a nearly straight propagation path across the interface. However, despite the optimized geometric contact, excessive tensile deformation introduces significant lattice stretching and interfacial stress concentration. The resulting modification of interatomic force constants enhances phonon scattering and weakens phonon transmission across the interface. Therefore, the interfacial thermal conductance decreases beyond the critical strain, indicating that the thermal transport is governed by the competition between geometric contact improvement and strain-induced phonon scattering [54].

**Figure 5:**
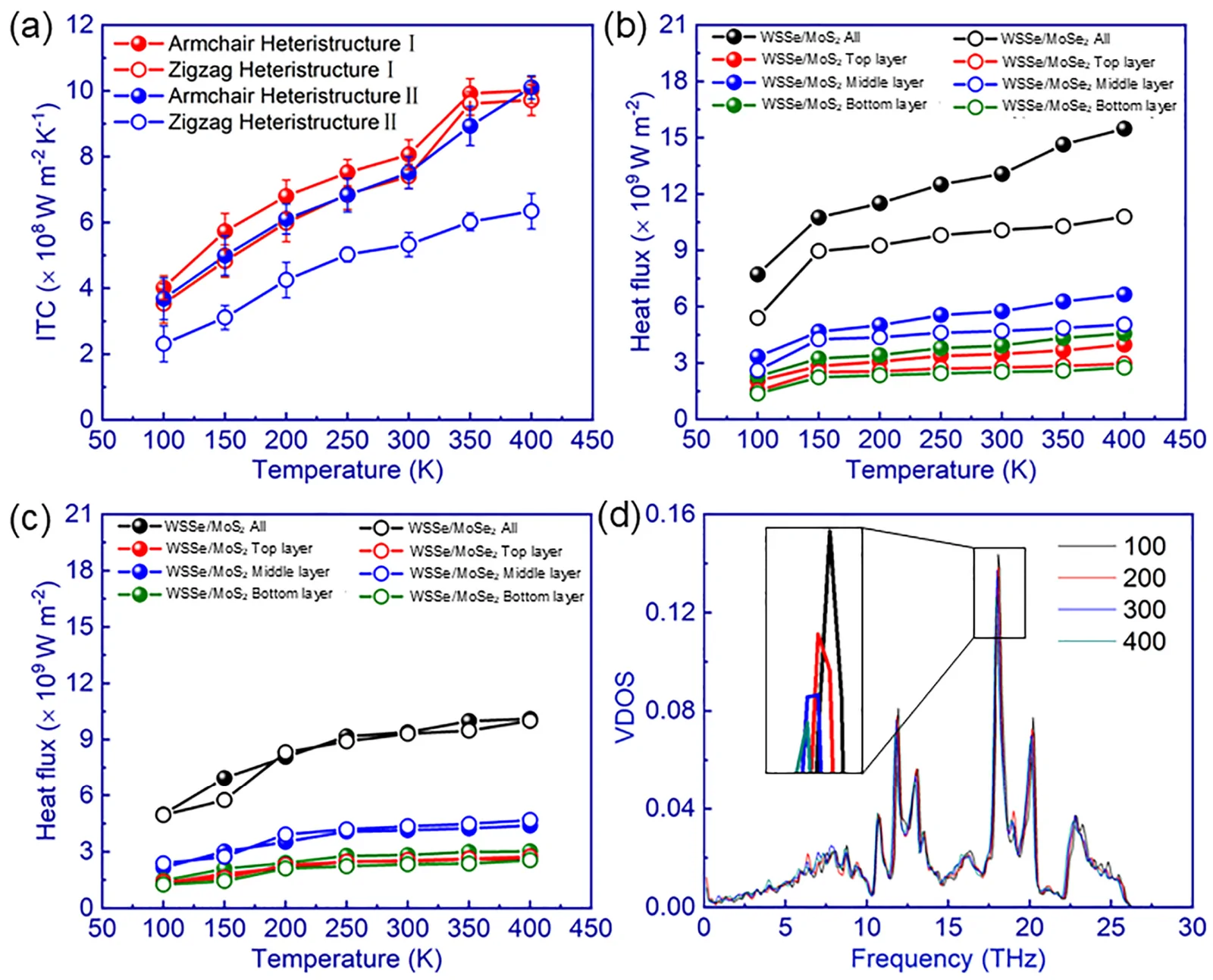
(**a**) The calculated interfacial thermal conductance of Armchair and Zigzag WSSe/MoX_2_ heterostructures obtained by temperatures ranging from 100 to 400 K. The calculated heat flux of different layers in (**b**) Armchair WSSe/MoS_2_ and (**c**) Zigzag WSSe/MoS_2_ heterostructures at temperatures ranging from 100 to 400 K. (**d**) Vibrational dynamic density of states of the WSSe/MoS_2_ heterostructure at different temperatures.

The temperature dependence of the thermal conductance across the intrinsically curved interface was further investigated. As shown in Figure 5a, the interfacial thermal conductance of WSSe/MoX_2_ heterostructures increases monotonically with temperature from 100 to 400 K. Obviously, the Armchair heterostructure exhibit consistently higher ITC values than their Zigzag counterparts throughout the investigated temperature range. Specifically, the ITC increases rapidly at low temperatures and gradually approaches a saturation trend at elevated temperatures. This behavior can be attributed to the enhanced excitation of heat-carrying phonon modes with increasing temperature, which promotes phonon coupling and interfacial energy transfer. Nevertheless, due to the classical nature of MD simulations, the low-temperature results should be regarded as qualitative trends because quantum statistics are not explicitly considered [46]. At high temperatures, the growth rate of ITC becomes less pronounced because phonon–phonon scattering intensifies, partially offsetting the increase in available phonon states [50]. It should be noted that the present NEMD simulations are based on classical MD, which assumes a classical phonon population following the equipartition theorem. At relatively low temperatures (such as 100 K), quantum effects may become important, and high-frequency phonon modes can be overpopulated compared with the real phonon distribution. Therefore, the calculated ITC values at low temperatures should be interpreted with caution, mainly reflecting qualitative variations in phonon coupling and interfacial heat-transfer mechanisms rather than exact quantitative predictions. Similar limitations of classical MD in low-temperature thermal transport simulations have been widely discussed in previous studies [10,55,56,57].

To gain further insight into the heat-transfer pathways, the layer-resolved heat fluxes of the Armchair and Zigzag heterostructures are presented in Figure 5b and c, respectively. The top, middle and bottom layers of the WSSe/MoX_2_ heterostructure are demonstrated as Figure 1a. One can see that the total heat flux increases continuously with temperature, indicating enhanced thermal energy transport at elevated temperatures. The middle layer contributes the largest fraction of heat flux, followed by the top and bottom layers, which reveals that heat conduction is primarily concentrated within the central transport channel, where stronger interlayer interactions facilitate more efficient phonon propagation [55]. Furthermore, Armchair WSSe/MoS_2_ heterostructure exhibits significantly higher heat flux than WSSe/MoSe_2_ across the entire temperature range, which is consistent with its larger ITC values in Figure 5a. Shown as Figure 5c, the overall heat flux also increases with temperature, confirming that thermal activation enhances phonon-mediated heat transport regardless of interfacial orientation. However, the magnitude of heat flux in the Zigzag structures is generally lower than that of the corresponding Armchair configuration. This reduction suggests that the zigzag interface possesses less efficient phonon transmission channels, leading to stronger phonon scattering and a larger thermal resistance. Similar to the Armchair configuration, the middle layer remains the dominant contributor to heat conduction, indicating that the fundamental heat-transfer mechanism is preserved despite the difference in crystallographic orientation.

To explore the microscopic origin of the temperature-dependent thermal transport behavior, Figure 5d presents the vibrational density of states of the WSSe/MoS_2_ heterostructure at temperatures ranging from 100 to 400 K. The major phonon peaks remain located within similar frequency ranges, indicating that the overall lattice vibrational characteristics are maintained with increasing temperature. However, noticeable peak broadening and intensity reduction can be observed at elevated temperatures, particularly in the low-frequency region highlighted in the inset. These changes are characteristic of enhanced anharmonic lattice vibrations and stronger phonon–phonon interactions. Meanwhile, the low-frequency acoustic phonon modes exhibit improved activation as temperature increases, providing additional channels for interfacial energy transfer. Since low-frequency phonons generally possess longer mean free paths and contribute significantly to heat transport across heterogeneous interfaces, their increased participation leads to the continuous enhancement of ITC observed in Figure 5a. Therefore, the temperature-induced increase in interfacial thermal conductance can be attributed to the combined effects of enhanced phonon population, improved phonon spectral overlap, and strengthened low-frequency vibrational coupling between the WSSe and WS_2_ layers. Overall, these results demonstrate that temperature plays a crucial role in regulating interfacial heat transfer in WSSe/MoS_2_ heterostructures by modifying phonon excitation, heat flux distribution, and vibrational coupling, while the armchair configuration consistently provides more efficient thermal transport than the zigzag configuration.

**Figure 6:**
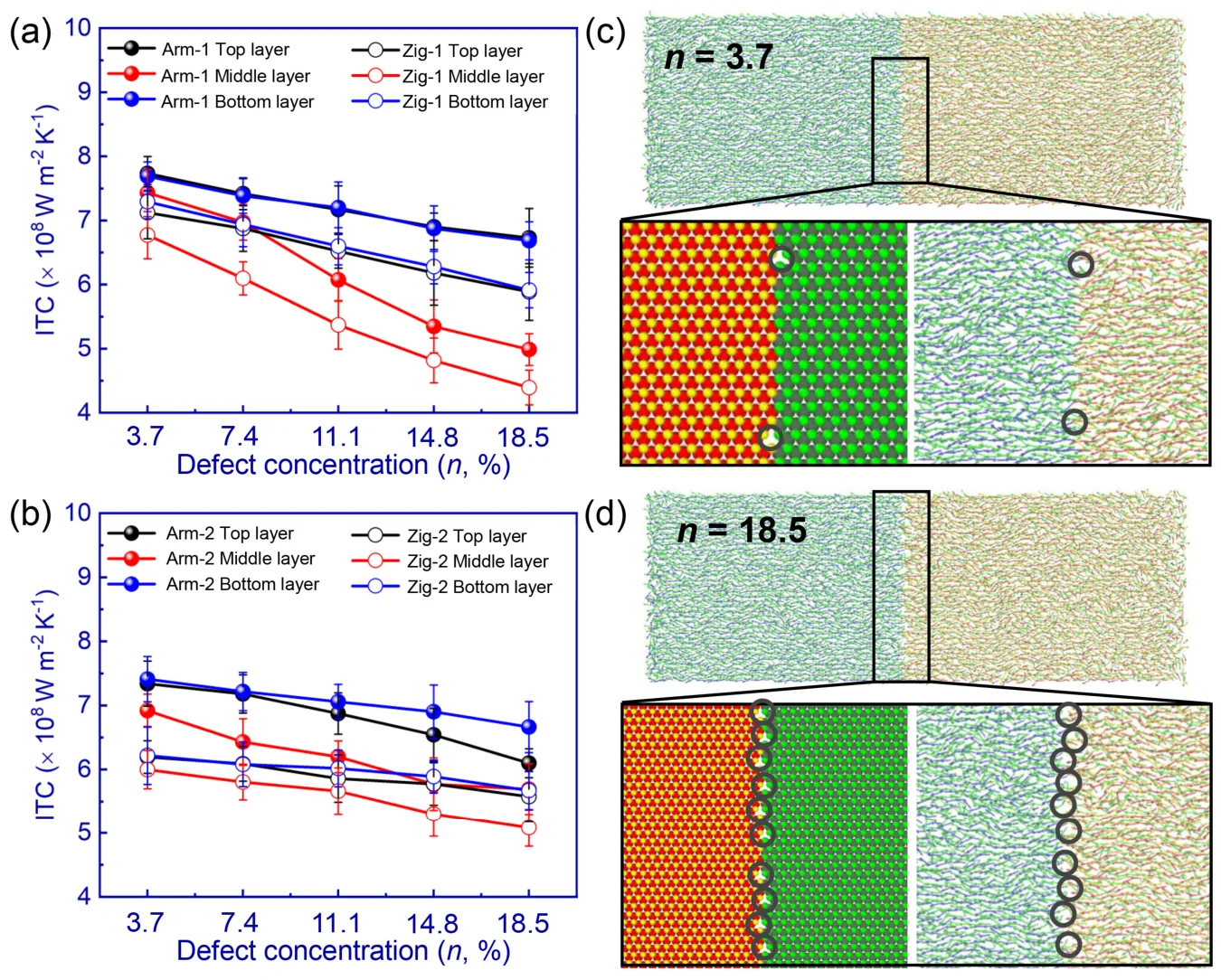
The response of interfacial thermal conductance on the number of vacancy defects introduced at the interface of the (**a**) WSSe/MoS_2_ and (**b**) WSSe/MoSe_2_ heterostructures. The calculated atomic heat flux vector distributions of the Armchair WSSe/MoS_2_ heterostructure with (**c**) *n* = 3.7 and (**d**) *n* = 18.5 vacancy defects introduced into the middle layer adjacent to the interface.

The dependence of the interfacial thermal conductance on the number of vacancy defects introduced near the interface for the WSSe/MoS_2_ and WSSe/MoSe_2_ heterostructures are addressed. The vacancy defects are randomly introduced into the selected interfacial atomic layers, and the defect concentration (*n*) is defined as the ratio of the number of removed atoms to the total number of atoms in the interface. As shown in Figure 6a,b, the ITC decreases monotonically with increasing vacancy concentration for both heterostructures, indicating that interfacial vacancies inevitably deteriorate phonon-mediated heat transport. Regardless of the interface configuration, vacancies introduced into the middle atomic layer adjacent to the interface produce the largest reduction in ITC, whereas defects located in the top and bottom layers have relatively weaker influences. This behavior demonstrates that the atomic layer directly participating in interfacial bonding dominates the vibrational coupling between the two monolayers. The introduction of vacancies in this critical region interrupts the continuity of interfacial bonding, weakens the interlayer force interactions, and consequently suppresses phonon transmission across the interface. Furthermore, the degradation of ITC is consistently more pronounced in the WSSe/MoS_2_ heterostructure than in the WSSe/MoSe_2_ counterpart, suggesting that the stronger interfacial coupling in WSSe/MoS_2_ is more sensitive to lattice imperfections.

The microscopic heat-transfer mechanism is further illustrated by the atomic heat flux vector distributions shown in Figure 6c,d. At a low vacancy concentration (*n* = 3.7), the heat flux remains largely continuous across the interface, with only slight local distortions around the defective sites, indicating that phonon transport is only weakly perturbed. As the vacancy number increases to *n* = 18.5, pronounced discontinuities and deflections of the heat flux vectors emerge near the interface. The vacancies act as efficient phonon-scattering centers, forcing heat carriers to bypass the defective regions and reducing the continuity of the heat-transfer pathways. Meanwhile, the removal of interfacial atoms decreases the number of available phonon transmission channels and modifies the local interatomic force constants, leading to enhanced phonon localization and reduced spectral overlap across the interface. The combined effects of bond discontinuity, intensified phonon scattering, and weakened vibrational coupling ultimately result in a continuous reduction in interfacial thermal conductance with increasing vacancy concentration [58].

Beyond fundamental understanding of phonon transport, the intrinsic curvature-regulated thermal transport mechanism revealed in this work may provide practical opportunities for advanced thermal management applications. Janus TMD heterostructures have been considered promising candidates for next-generation flexible electronics, optoelectronic devices and nanoscale energy conversion systems due to their unique electronic properties, structural flexibility and interfacial tunability. In these applications, thermal resistance at heterogeneous interfaces critically affects device efficiency, reliability and lifetime. Therefore, the ability to actively regulate interfacial thermal conductance through intrinsic interface curvature and external mechanical stimuli may provide a novel pathway for developing adaptive thermal management materials and high-performance 2D devices.

## 4. Conclusions

The main conclusions of this study are summarized as follows: (1) The effect of intrinsic curved interfaces on interfacial thermal transport in Janus TMD heterostructures was systematically investigated, revealing that interface curvature introduces structural modulation and provides an additional degree of freedom for tuning thermal boundary conductance. (2) The coupling relationship between interfacial curvature, atomic-scale structural characteristics and phonon transport behavior was clarified. The results demonstrate that curvature-induced structural changes can modify vibrational coupling and phonon transmission across the interface, thereby regulating interfacial heat transfer. (3) This work highlights the potential of intrinsic curved interfaces as an effective strategy for phonon engineering in two-dimensional heterostructures and provides new insights into the design of advanced thermal management materials with controllable interfacial thermal transport properties.

## Data Availability

The data presented in this study are available upon request from the corresponding author.

## Abbreviations

2D	Two-dimensional
TMDs	transition metal dichalcogenides
ITC	interfacial thermal conductance
CVD	chemical vapor deposition
MD	molecular dynamics
NEMD	non-equilibrium molecular dynamics
SW	Stillinger–Weber
VDOS	vibrational density of states
*ε*	strain
